# Integrated Analysis of Multiple Databases Identifies Tissue Inhibitor of Metalloproteinase 1 Expression and Its Association with the Immune Microenvironment in Colorectal Cancer

**DOI:** 10.3390/genes17080977

**Published:** 2026-08-20

**Authors:** Yun Xie, Jun Li, Zuwei Yan, Wenguang Zhang

**Affiliations:** 1 Faculty of Science, Inner Mongolia Agricultural University, Hohhot 010018, China; xieyunhust@163.com; 2 School of Life Science and Technology, Inner Mongolia University of Science and Technology, Baotou 014010, China; 3 Inner Mongolia Engineering Research Center of Genomic Big Data for Agriculture, Inner Mongolia Agricultural University, Hohhot 010018, China; 4 Institute for Rural Revitalization, Inner Mongolia Agricultural University, Hohhot 010018, China

**Keywords:** colorectal cancer, *TIMP1*, tumor immune microenvironment, prognostic biomarker, extracellular matrix

## Abstract

Background: In recent decades, the incidence of colorectal cancer (CRC) has been rising worldwide. CRC ranks second in cancer-related mortality. The identification of reliable biomarkers for early diagnosis and prognosis prediction, along with a deeper understanding of the underlying molecular events, holds substantial promise for improving patient outcomes. The tissue inhibitor of the metalloproteinase 1 (*TIMP1*) gene is overexpressed in various gastrointestinal malignancies and contributes to tumor progression. However, its role in regulating the CRC tumor immune microenvironment (TIME) and its potential as a clinically actionable prognostic biomarker remain unclear. Methods: To probe how *TIMP1* acts as a prognosis-related candidate biomarker in colorectal carcinoma, TCGA-derived datasets were adopted to conduct Kaplan–Meier survival assessment. We also investigated the connection between the expression abundance of *TIMP1* and the infiltration of immune populations and intratumoral lymphocytes; furthermore, immune checkpoint-related genes were systematically assessed across multiple tumor types via the TISIDB and TIMER2.0 platforms, with particular emphasis on CRC. We adopted the ESTIMATE scoring system to figure out how *TIMP1* gene expression correlates with the phenotypic properties of the colorectal-cancer TIME. We relied on the limma toolkit for the screening of differential transcripts from high-*TIMP1* and low-*TIMP1* cohorts. Enrichment assessments covering Gene Ontology terms and Kyoto Encyclopedia of Genes and Genomes entries were then carried out to predict the potential biological pathways associated with *TIMP1*. We constructed the protein–protein interaction map for *TIMP1*-interacting partners via the STRING repository. To further explore *TIMP1*-correlated genes, we performed Venn diagram intersection analysis combined with Spearman’s correlation test. Finally, quantitative reverse-transcription PCR was then implemented to detect *TIMP1* messenger-RNA abundance inside the RKO colorectal carcinoma cell line as well as normal colonic epithelial CCD-18Co cells, which offered in vitro experimental verification for our bioinformatic outcomes. Results: According to outcome data, *TIMP1* transcripts were markedly up-regulated in CRC specimens and cell lines relative to normal samples. Elevated *TIMP1* expression served as a poor-prognosis indicator for overall survival (hazard ratio [HR] = 0.43, 95% confidence interval [CI] = 0.29–0.64, *p* < 0.001) and disease-specific survival (HR = 0.39, 95% CI = 0.22–0.68, *p* = 0.001) among colorectal-carcinoma patients. *TIMP1*-high and *TIMP1*-low groups exhibited notable differences in immune cell infiltration (CD8^+^ T, macrophage, mast, neutrophil, B, monocyte, dendritic, and CD4^+^ T cells). *TIMP1* expression was also significantly correlated with tumor-infiltrating lymphocytes, key immune checkpoint genes (e.g., *CD274* [*PD-L1*] and *CTLA4*), and immunomodulatory chemokines (e.g., *CCL3* and *CCL5*). Twelve *TIMP1*-interacting DEGs were selected: *COL5A1*, *FN1*, *PRG4*, and a cluster of nine *MMPs* (*MMP1*/*2*/*3*/*7*/*8*/*9*/*11*/*13*/*14*), all of which showed significant positive correlations with *TIMP1* (r = 0.31–0.63, all *p* < 0.001). Conclusions: *TIMP1* expression correlates with features of the tumor immune microenvironment and extracellular matrix remodeling in CRC, suggesting that *TIMP1* shows potential as a candidate biomarker. However, its potential as a therapeutic target warrants further experimental investigation.

## 1. Introduction

### 1.1. Background

As one of the most prevalent malignant tumors across the globe, colorectal carcinoma (CRC) constitutes the second-most common source of cancer-associated fatalities worldwide [1]. The global annual incidence is projected to reach 2.5 million new cases by 2035 [2]. Despite advances in screening programs facilitating earlier tumor detection, around one-quarter of colorectal-carcinoma patients are diagnosed with late-stage lesions. Moreover, 25–50% of individuals receiving treatment for localized lesions will later develop distant tumor spread [3]. These population-based clinical realities demonstrate that researchers urgently need to unravel CRC-related molecular foundations and screen for robust biomarkers, which serve for timely detection and refined prognosis classification.

Tissue inhibitor of metalloproteinase 1 (*TIMP1*) primarily inhibits matrix metalloproteinases (MMPs), thereby regulating extracellular matrix (ECM) remodeling and degradation [4,5]. Lots of research have uncovered dysregulated *TIMP*1 expression in an extensive range of human pathological conditions, which include gastric carcinoma [6], lung adenocarcinoma [7], Alzheimer’s disease [8], heart-muscle infarction [9], breast cancer [10], as well as ovarian cancer [11]. Accumulating evidence suggests that *TIMP1*-enriched extracellular vesicles may contribute to distant metastasis by promoting ECM remodeling in CRC [12]. Moreover, gradual elevation of *TIMP*1 abundance can be observed during disease progression, spanning ulcerative colitis-related inflammation to advanced metastatic CRC [13], and is a ferroptosis-associated biomarker for ulcerative colitis [14]. At the transcriptional level, *TIMP1* is subject to complex regulatory mechanisms, including competitive promoter binding, cytosolic DNA sensing pathways, cellular responses to citrate cycle perturbations, and transcriptional repression mediated by ultraviolet A and β-alanine [15]. Clinically, elevated *TIMP1* mRNA levels have been linked to more advanced CRC stages [16], and increased circulating *TIMP1* concentrations have been associated with poorer survival outcomes in CRC patients [17,18].

### 1.2. Rationale and Knowledge Gap

The relevance of *TIMP1* in CRC has previously been established; however, systematic investigations into its comprehensive biological functions, particularly its role in modulating the immune microenvironment within tumor lesions (TIME), remain limited. The TIME comprises stromal cells, immune cells, and various soluble mediators, and is closely linked to both CRC development and the efficacy of immunotherapeutic strategies [19]. Thus, understanding *TIMP1* interactions with the TIME could reveal novel therapeutic targets.

### 1.3. Objective

We attempted to clarify whether *TIMP1* acts as a promising prognostic biomarker within the population of colorectal-cancer sufferers and its functional role in tumor immunomodulation by: (a) characterizing *TIMP1* expression patterns across clinical cohorts and cell lines, (b) assessing their association with patient survival, (c) examining their correlation with tumor-infiltrating immune cell abundance and immune checkpoints, and (d) deciphering the underlying mechanisms linking *TIMP1* to TIME remodeling and ECM metabolism. Through these integrated analyses, we aim to assess the prognostic value of *TIMP1* in CRC and its association with immune infiltration, with potential implications for clinical management.

## 2. Methods

### 2.1. Research Materials

Two cell strains, namely the human normal colonic epithelial CCD-18Co and colon adenocarcinoma RKO, were acquired from the Shanghai-based Chinese Academy of Sciences Cell Bank. Cell authentication relied on short tandem-repeat fingerprinting, and zero *Mycoplasma* infection was detected for every cultivated cell line.

Immune cell infiltration landscapes and transcriptional levels of *TIMP1* across assorted cancer subtypes were assessed with assistance from the TIMER 2.0 online database (http://timer.cistrome.org/) (accessed on 12 February 2024). Transcriptome sequencing raw data and matched clinical metadata from colorectal-disease cohorts, including colon adenocarcinoma (COAD) and rectal adenocarcinoma (READ) samples, were extracted from the TCGA data portal (https://portal.gdc.cancer.gov/) (accessed on 12 February 2024). These resources supported subsequent survival-statistic exploration and screening for differentially expressed genes. The GEPIA analysis platform (http://gepia.cancer-pku.cn/) (accessed on 12 February 2024), which aggregates RNA-sequencing information sourced from TCGA and the Genotype-Tissue Expression (GTEx) repositories, was applied to compare *TIMP1* transcriptional abundance in non-lesioned tissues and malignant specimens. Extra expression data concerning *TIMP1* within colorectal-cancer samples were gathered from datasets hosted on the Gene Expression Omnibus (GEO) repository (https://www.ncbi.nlm.nih.gov/geo/) (accessed on 12 February 2024) (GSE41657: *n* = 37, 25 CRC tissues vs. 12 normal tissues; GSE52060: *n* = 46, 23 CRC tissues vs. 23 normal tissues; GSE74602: *n* = 60, 30 CRC tissues vs. 30 normal tissues; GSE160988: *n* = 12, 6 CRC tissues vs. 6 normal tissues). Information concerning the intracellular positioning, spatial-protein deployment and immunohistochemical staining traits of *TIMP1* was acquired from the online-resource Human Protein Atlas (THPA; https://www.proteinatlas.org/) (accessed on 12 February 2024). Correlations between this gene and tumor-derived immune events inside colorectal carcinoma samples were mined with the TISIDB web-server (http://cis.hku.hk/TISIDB/) (accessed on 12 February 2024). This platform aggregates multi-source datasets covering tumor-infiltrating-lymphocyte populations (TILs), immune checkpoint regulators and chemokine mediators. For the exploration of intermolecular protein connections (PPI), raw interaction data were fetched from the STRING repository (https://string-db.org/) (accessed on 12 February 2024). Molecular pairs holding a confidence-index cutoff above or equal to 0.7 were screened, so as to generate a molecular-network diagram that takes *TIMP1* as the core hub gene.

### 2.2. Survival Analysis

Colorectal-cancer participants sourced from the TCGA dataset were separated into two cohorts based on the median transcript abundance of *TIMP1*, namely the *TIMP1*-high and *TIMP1*-low transcription subgroups. Survival disparities across the two subgroups were contrasted via Kaplan–Meier survival curves. The log-rank inspection was adopted to judge statistical-level discrepancies of overall survival (OS) and disease-specific survival (DSS). Single-factor Cox regression statistics were carried out for the computation of hazard ratios alongside their 95% confidence intervals. During Kaplan–Meier plotting and Cox proportional-hazard modeling, specimens with elevated *TIMP1* transcript levels were set as the benchmark cohort. A hazard-ratio value less than one stood for lowered death risk within the low-expression population relative to the benchmark high-expression subgroup. For further verification on the prognostic predictive capacity of *TIMP1*, time-dependent ROC-curve analysis was implemented. Corresponding AUC indices were computed to assess the 1-, 3- and 5-year predictive efficiency for OS and DSS outcomes.

### 2.3. TIMP1 Expression in Relation to Immune Features in CRC

Immune infiltration profiles in CRC were examined using the TIMER 2.0 platform. We carried out correlation analysis between the gene-expression status of *TIMP1* and the enrichment degree of twelve immune cell subgroups; typical examples covering macrophages, neutrophils and T cell populations, and Spearman’s correlation coefficients were calculated. The ESTIMATE algorithm was applied to TCGA CRC data to compute the stromal, immune, and ESTIMATE scores (combined stromal and immune scores). Pearson’s correlation coefficients were applied to evaluate the association with *TIMP1* expression. Correlations between *TIMP1* expression and 28 TILs in CRC were evaluated using the TISIDB platform, relevant-variable comparisons were carried out, and Spearman rank-order correlation testing served to screen statistically significant outcomes. Pearson correlation computation was adopted to probe the linkage between *TIMP*1 transcription level and 46 widely studied immune checkpoint molecules (including, *CD274*, *TIGIT*, and *CTLA4*) and immunomodulatory chemokine (including, *CCL3*, *CCL5*, *CXCL8*, and *CXCL10)* genes in TCGA CRC data.

### 2.4. Functional-Enrichment Analysis

The R-based limma software package was utilized to screen out divergent transcripts between subgroups with high- and low-level *TIMP*1 transcription within the TCGA-CRC dataset (R version 4.2.2). Genes satisfying the screening benchmark of *p*-value (*P_adj_* < 0.05) and |log_2_FoldChange| > 1 were regarded as candidate differential-expression markers. Raw TCGA RNA-seq count data were pre-processed using the voom pipeline from the R-based limma software package. Genes with low expression were filtered out prior to analysis. The voom function was then applied to transform the filtered count data to log_2_-counts-per-million (logCPM) and to calculate observational-level precision weights, which model the mean-variance relationship inherent in RNA-seq count data. These normalized logCPM values and their associated weights were subsequently used for differential expression analysis with the lmFit and eBayes functions from the limma package. We adopted Euclidean distance together with Ward.D2 linkage criteria to carry out hierarchical clustering for screened differential genes, and the clustering computation covered both gene and sample data. The visualized heatmap figure was produced via the pheatmap toolkit under the R 4.2.2 software environment. All functional-enrichment work was completed with the help of the clusterProfiler R-package (version 4.2.2). The enrichGO function was utilized to explore functional terms of up-regulated differential transcripts. The Benjamini–Hochberg algorithm eliminated bias from repeated comparisons, and Storey’s approach calculated q-values for false-discovery-rate regulation; items with a q-value below 0.05 were regarded as statistically meaningful. For KEGG-pathway exploration, the enrichKEGG tool was applied, and outcomes passing the BH-corrected-*p*-value cutoff (*P_adj_* < 0.05) were recognized as significant.

### 2.5. Ppi Network Construction and Core Gene Selection

*TIMP1*-interacting proteins were retrieved from the STRING platform (confidence score ≥ 0.7). Overlap analysis was performed to identify genes shared between *TIMP1*-interacting proteins and *TIMP1*-associated DEGs. We explored the association between *TIMP-1* and these screened candidate biomarkers based on the TCGA colorectal-cancer dataset. Spearman rank-order correlation analysis was adopted for gene-expression comparison, with *p*-value less than 0.05 regarded as the threshold for statistically significant outcomes.

### 2.6. Cell Culture and Quantitative Real-Time Pcr (Rt-Qpcr)

The CCD-18Co and RKO cell strains were cultivated within Dulbecco-modified Eagle’s medium supplemented with 10% fetal bovine serum together with 1% penicillin–streptomycin mixed solution. The incubator environment was kept at 37 °C and 5% carbon-dioxide concentration. Total cellular-RNA was isolated using RNAiso Plus (Code No. 9108). and reverse transcription for cDNA synthesis was performed using the PrimeScript RT Reagent Kit (both from Takara Bio Inc., Kusatsu, Shiga, Japan). Subsequent quantitative real-time PCR was carried-out on QuantStudio 5 equipment (Thermo Fisher Scientific, Waltham, MA, USA), with Takara-supplied SYBR Green I Master Mix serving as the reaction mixture. *TIMP1*-specific primers (forward: 5′-GTTGTTGCTGTGGCTGATAG-3′; reverse: 5′-TGTGGGACCTGTGGAAGTA-3′) were obtained from Sangon Biotech (Shanghai, China), with GAPDH as the reference. Relative *TIMP1* mRNA levels were determined by the 2^−ΔΔCt^ method.

### 2.7. Statistical Analysis

All related data processing and statistical computation were completed with the R-language platform (version 4.2.2), and GraphPad Prism 9.0 software was adopted for drawing statistical diagrams. For non-normally distributed data, differences in *TIMP1* expression across diverse sample types were evaluated with Kruskal–Wallis non-parametric inspection. The Shapiro–Wilk test was firstly utilized to check data normality, and then paired-group disparities between tumor and adjacent-normal tissues were analyzed with Student’s *t*-test. Stage-specific expression differences were examined via the Wilcoxon test for variables with non-normal distributions.

## 3. Results

### 3.1. Timp1 Is Significantly Overexpressed in Crc

**Figure 1:**
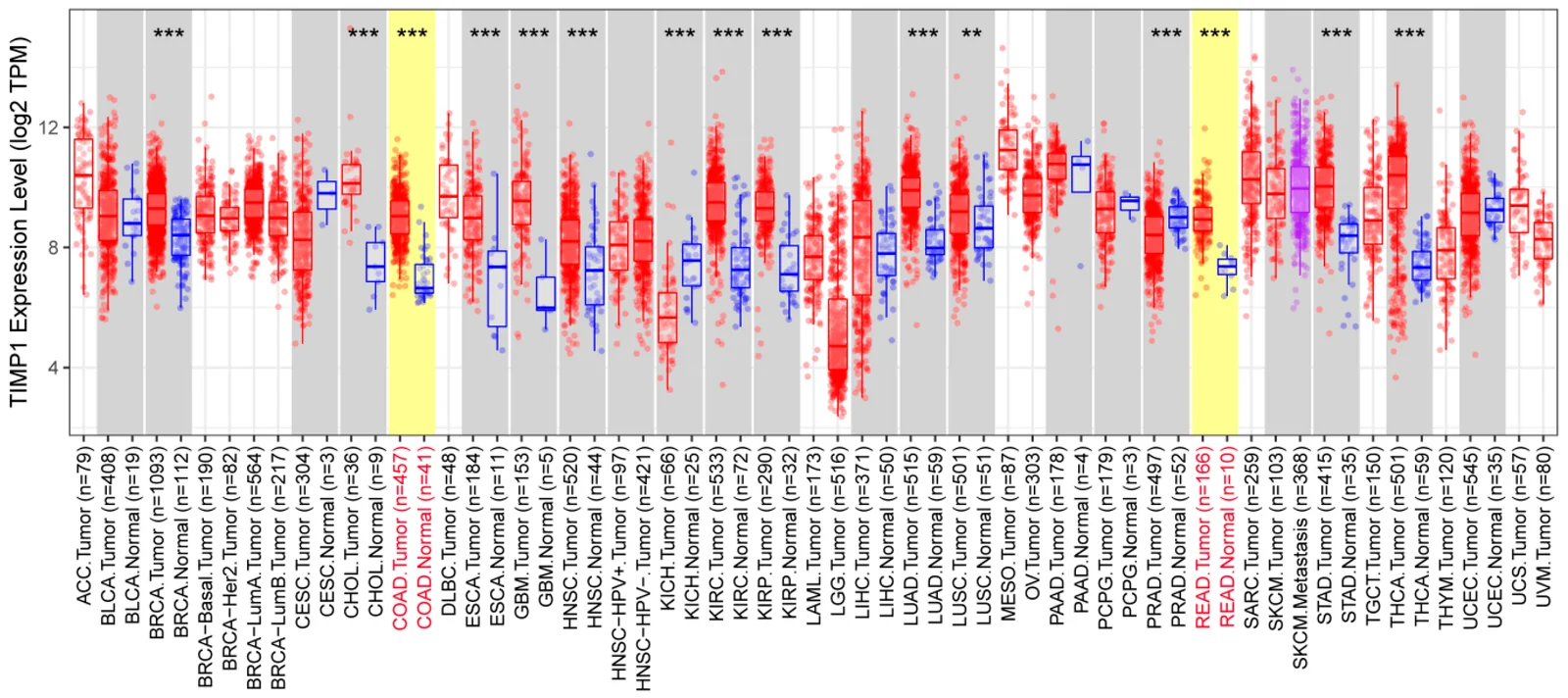
Expression profiles of *TIMP1* in human tumors and normal tissues (TIMER version 2.0). *** *p* < 0.001, ** *p* < 0.01.

**Figure 2:**
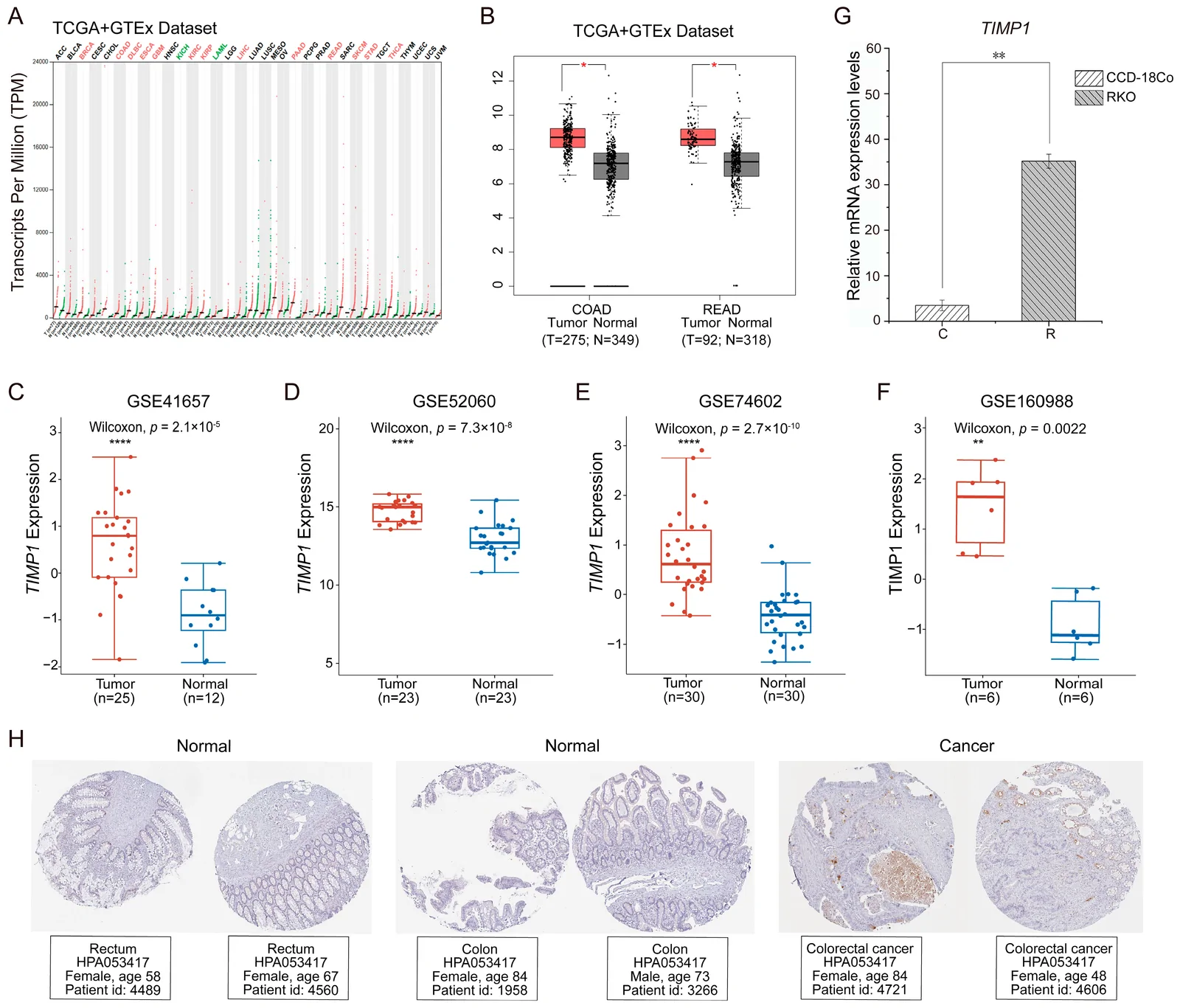
*TIMP1* overexpression in CRC tissues and cell lines. (**A**) Pan-cancer *TIMP1* expression pattern (TCGA + GTEx dataset, GEPIA database). (**B**) *TIMP1* expression differences between cancerous and normal colon-rectal samples in COAD and READ (TCGA + GTEx dataset, GEPIA database). (**C**–**F**) *TIMP1* expression in CRC tissues vs. normal tissues in GEO datasets: GSE41657 (**C**), GSE52060 (**D**), GSE74602 (**E**), and GSE160988 (**F**). (**G**) Relative *TIMP1* mRNA abundance in CRC (RKO) and normal colon epithelial (CCD-18Co) cell lines (quantitative real-time PCR validation). (**H**) Immunohistochemical-based detection for *TIMP1* protein within colorectal-carcinoma and adjacent-healthy specimens (Human Protein Atlas repository). **** *p* < 0.0001, ** *p* < 0.01, * *p* < 0.05.

Analysis of TIMER 2.0 data revealed that *TIMP1* was significantly overexpressed in 15 tumor types, including COAD and READ, compared with that in normal tissues (Figure 1). We observed the up-regulated transcription level of *TIMP1* in colon and rectal adenocarcinoma specimens against their matched healthy counterparts via GEPIA datasets (Figure 2A,B). Consistent results were obtained from four independent GEO datasets: GSE41657 (*p* < 0.001), GSE52060 (*p* < 0.001), GSE74602 (*p* < 0.001), and GSE160988 (*p* = 0.002) (Figure 2C–F). RT-qPCR validation revealed that *TIMP1* mRNA levels were significantly higher in RKO CRC cells than those in CCD-18Co normal colon epithelial cells (Figure 2G). With immunohistochemical datasets obtained from the THPA platform, we verified the higher-level *TIMP1* protein abundance inside colorectal tumor samples compared to non-cancerous tissues (Figure 2H).

### 3.2. TIMP1 Shows Potential as a Candidate Biomarker for CRC

**Figure 3:**
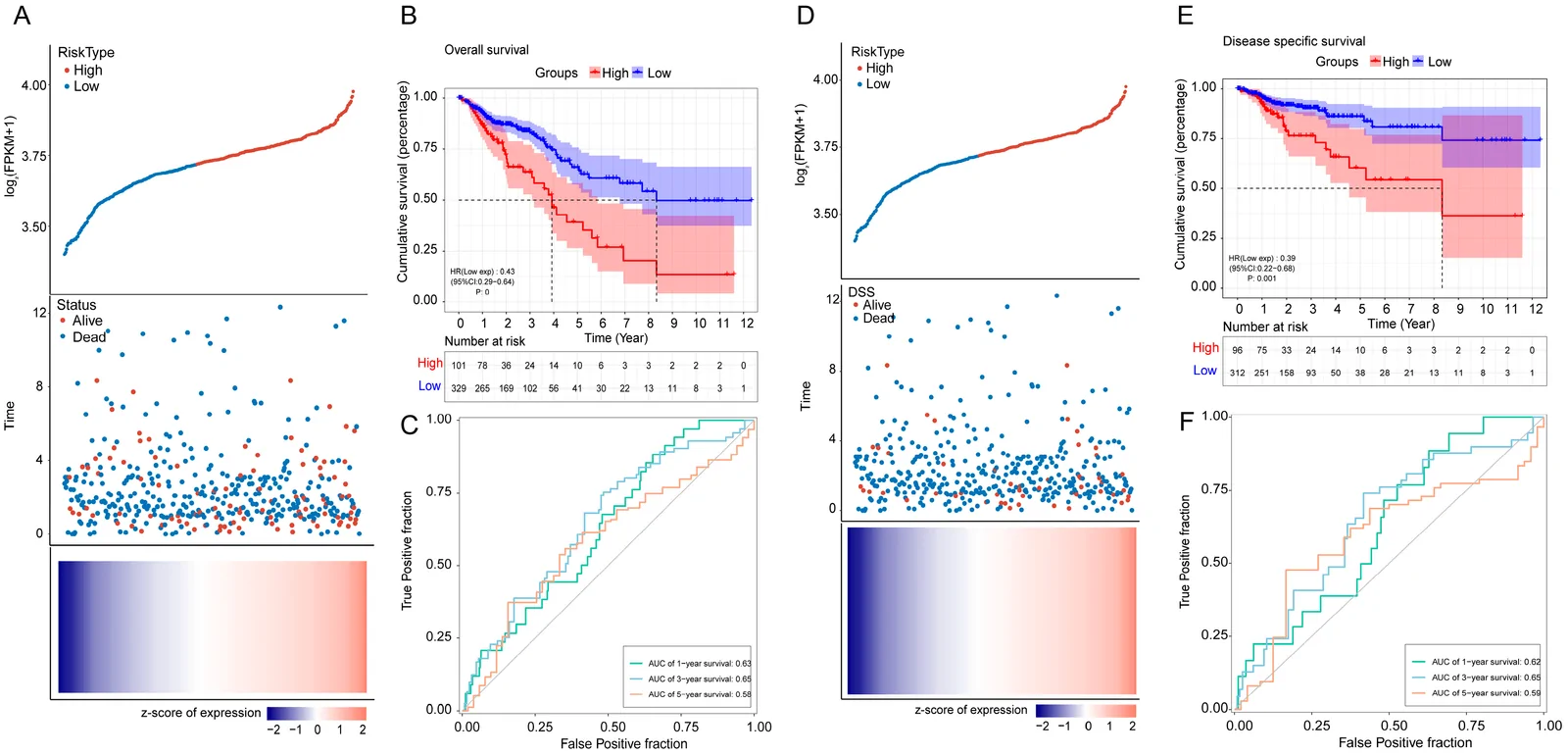
Prognostic value of *TIMP1* in CRC. (**A**) Messenger-RNA distribution data, survival status records and gene-expression heatmap of the target gene. (**B**) Overall-survival Kaplan–Meier survival-trajectory diagrams. (**C**) Time-dependent receiver-operating-characteristic curves based on OS data. (**D**) Survival-outcome exploration of *TIMP1* for DSS. (**E**) Kaplan–Meier-based DSS lines. (**F**) ROC plots with time-dependent parameters for DSS. (The high-expression group was defined as the reference group.)

Shorter survival durations of OS and DSS were detected among CRC subjects with up-regulated *TIMP1*. The corresponding prognostic indexes were OS (HR = 0.43, 95% CI = 0.29–0.64, *p* < 0.001) and DSS (HR = 0.39, 95% CI = 0.22–0.68, *p* = 0.001), compared to cases with weak *TIMP1* expression (Figure 3A–D). ROC curve analysis demonstrated that *TIMP1* had moderate prognostic predictive value for 1-, 3-, and 5-year OS (AUC = 0.63, 0.65, and 0.58, respectively) and DSS (AUC = 0.62, 0.65, and 0.59, respectively) (Figure 3C,F). However, no marked difference in *TIMP1* expression was detected across CRC samples at different T stages (Figure S1A), nor among different pathological stage OS (Figure S1B).

### 3.3. TIMP1 Expression Correlates with Immune Cell Infiltration and Time Remodeling

**Figure 4:**
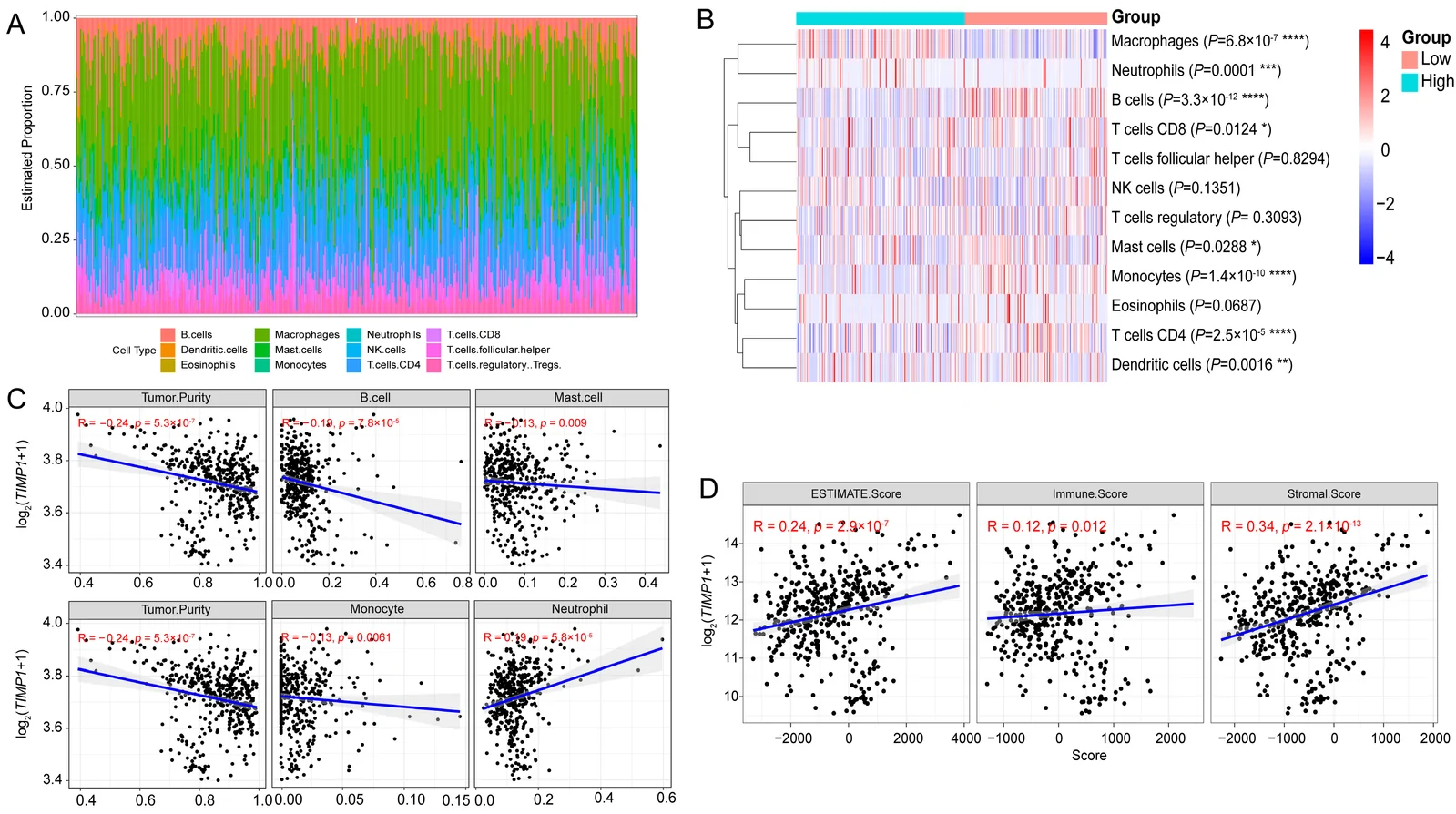
Association of *TIMP1* expression with immune infiltration in CRC. (**A**) Immune cell-type composition stratified by tumor origin. (**B**) Heatmap of score-based correlations between *TIMP1* and immune infiltrates. (**C**) *TIMP1* expression versus tumor purity and four specific immune cell types (B cells, mast cells, monocytes, neutrophils). (**D**) Associations of *TIMP1* with ESTIMATE, immune, and stromal scores. **** *p* < 0.0001, *** *p* < 0.001, ** *p* < 0.01, * *p* < 0.05.

An initial investigation was carried out to profile the relative quantities of multiple immune cell subgroups among CRC specimens (Figure 4). Analysis of immune cell infiltration showed that *TIMP1*-low tumors had significantly higher infiltration levels of the vast majority of immune cells than *TIMP1*-high tumors. Among them, the two subgroups displayed markedly different immune cell infiltration profiles for macrophages (*p* < 0.001), neutrophils (*p* < 0.001), B cells (*p* < 0.001), CD8^+^ T cells (*p* = 0.01), mast cells (*p* = 0.03), monocytes (*p* < 0.001), CD4^+^ T cells (*p* < 0.001), and dendritic cells (*p* = 0.002) (Figure 4B). Cluster analysis revealed that macrophages, neutrophils, B cells, monocytes, CD4^+^/CD8^+^ T cells, and dendritic cells clustered into the same major branch, with highly correlated infiltration patterns that were coordinately elevated in the *TIMP1*-low group, collectively forming the immune microenvironment signature of these tumors. In contrast, regulatory T cells, NK cells, and eosinophils clustered into another branch, with the two subgroups displaying comparable infiltration levels, indicating that the infiltration levels of these immune cells are less affected by *TIMP1* expression. The relevant association test uncovered that *TIMP1* transcript abundance presented favorable links to neutrophil immune penetration (r = 0.19, *p* < 0.001), ESTIMATE-derived indices (r = 0.24, *p* < 0.001), and immune-landscape marks (r = 0.12, *p* = 0.01) as well as stromal index values (r = 0.34, *p* < 0.001), as illustrated in Figure 4D. Conversely, its expression level held inverse relationships against tumor cellular purity (r = −0.24, *p* < 0.001), monocyte accumulation (r = −0.13, *p* = 0.006), mast cell immune infiltration (r = −0.13, *p* = 0.009) and B-lymphocyte penetration (r = −0.19, *p* < 0.001), which is displayed in Figure 4C.

### 3.4. TIMP1 Correlates with Tils, Immune Checkpoints, and Chemokines

**Figure 5:**
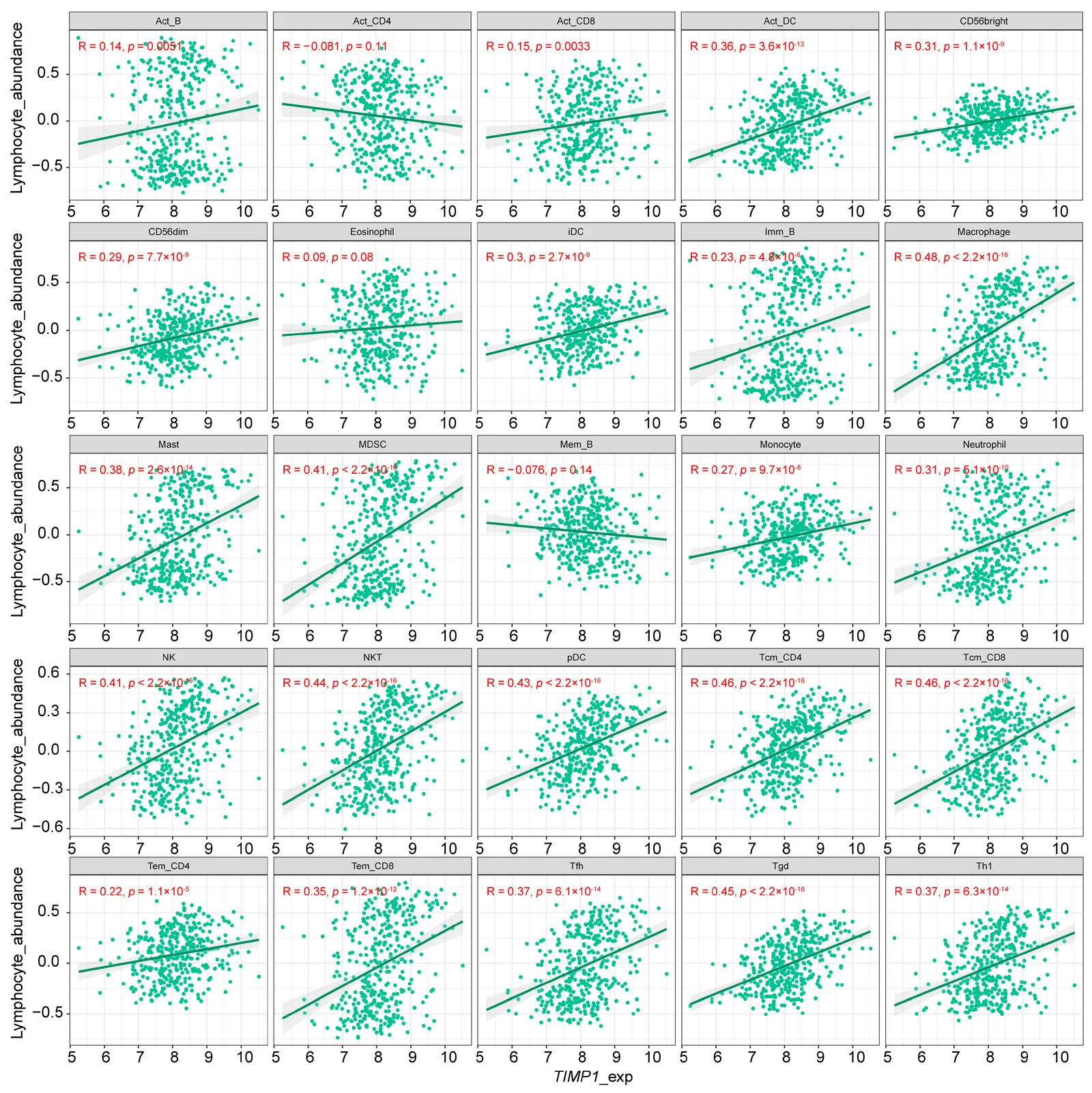
Association landscape of *TIMP1* mRNA levels with 25 distinct TILs that exhibited statistically meaningful correlations.

**Figure 6:**
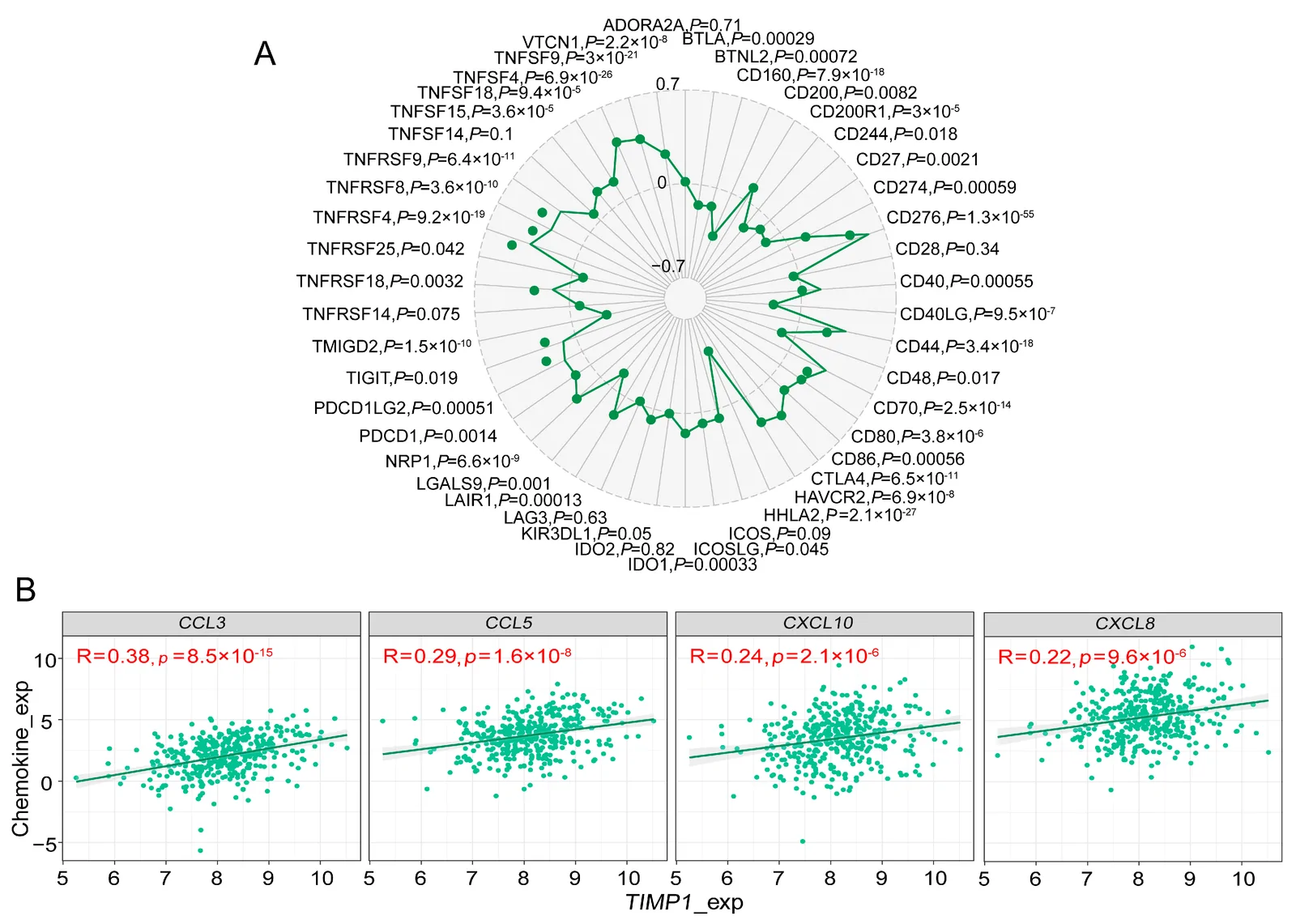
*TIMP1* correlation with immune checkpoint and chemokine genes in CRC. (**A**) Associations with common immune checkpoint genes. (**B**) Correlation with chemokine genes.

Interrogation of the TISIDB repository revealed that *TIMP1* transcript abundance in CRC is tightly linked to the abundance of multiple TILs. The strongest positive associations were observed for macrophages (r = 0.48, *p* < 0.001), followed by CD4^+^ central memory T cells (Tcm_CD4, r = 0.46) and CD8^+^ central memory T cells (Tcm_CD8, r = 0.46) (Figure 5). A systematic correlation scan against 46 canonical immune checkpoint genes indicated that 37 of these targets exhibited significant co-expression with *TIMP1*, including *CD274* (*PD-L1*, r = 0.32), *CTLA4* (r = 0.28), and *TIGIT* (r = 0.30) (Figure 6A). Furthermore, *TIMP1* mRNA levels positively paralleled the expression of immunomodulatory chemokines such as *CCL3* (r = 0.38), *CCL5* (r = 0.29), *CXCL8* (r = 0.22), and *CXCL10* (r = 0.24), all with *p* < 0.001 (Figure 6B).

### 3.5. Functional Enrichment of TIMP1-Associated Degs

**Figure 7:**
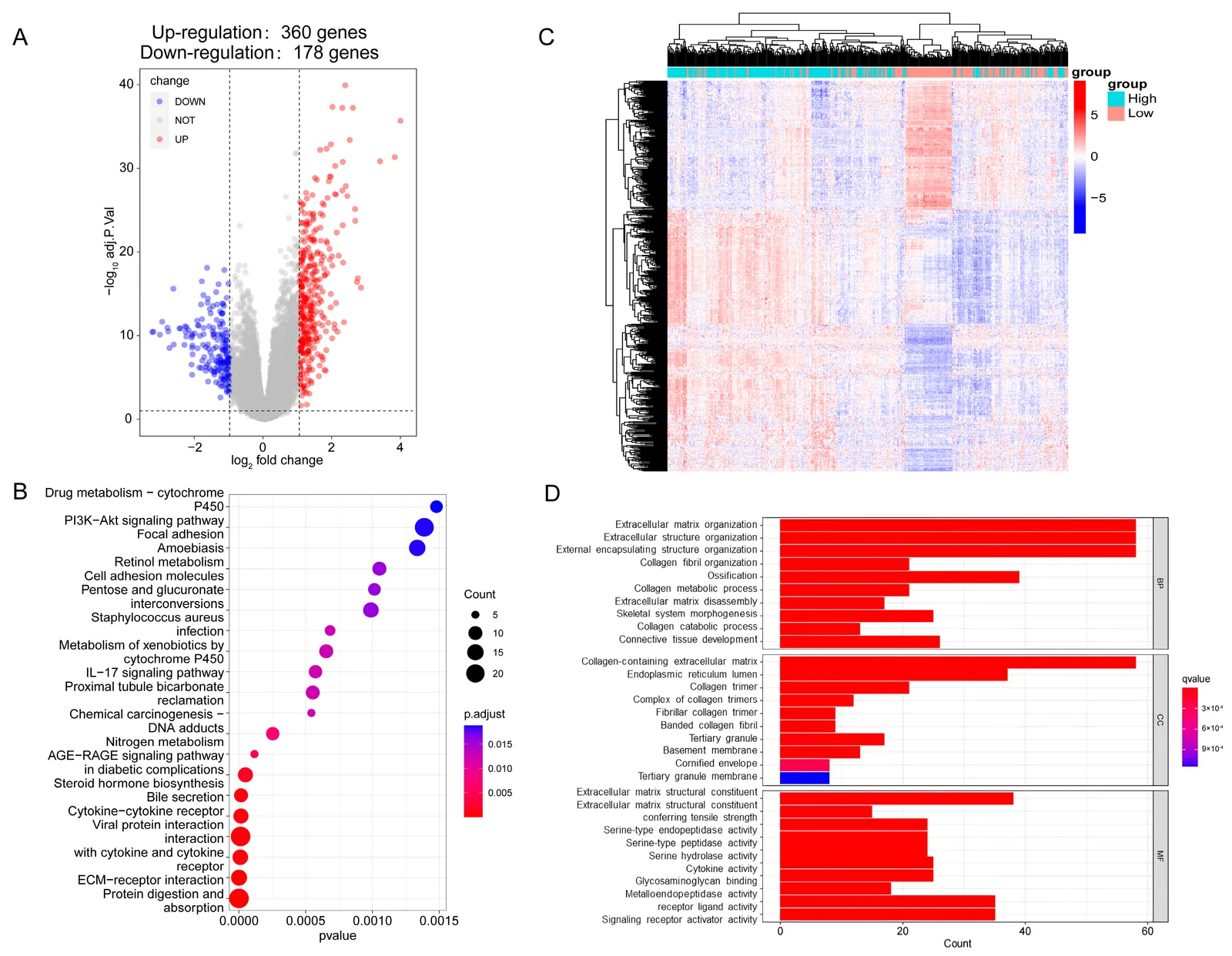
Functional enrichment of *TIMP1*-associated DEGs. (**A**) Volcano diagram displaying gene-level disparities in transcript abundance from high- and low-*TIMP1* subgroups. (**B**) A heatmap graphic visualizing the transcriptional profiles of these altered genes. Hierarchical grouping was implemented with Euclidean distance metrics alongside the Ward-D2 clustering algorithm. (**C**) Functional-enrichment examination within the KEGG database for genes with elevated transcription. (**D**) Gene Ontology enrichment statistics targeting these overexpressed gene candidates.

Stratification of CRC samples by *TIMP1* expression produced 538 DEGs (360 up-regulated and 178 down-regulated; Figure 7A,B). Functional annotation of the up-regulated genes (Figure 7C,D) concurrently highlighted KEGG pathways, including ECM-receptor interaction, PI3K-Akt signaling, focal adhesion, and IL-17 signaling, alongside GO biological processes related to extracellular structure organization, ECM organization, collagen-containing ECM, and receptor ligand activity.

### 3.6. Core Genes Interacting with TIMP1 in CRC

**Figure 8:**
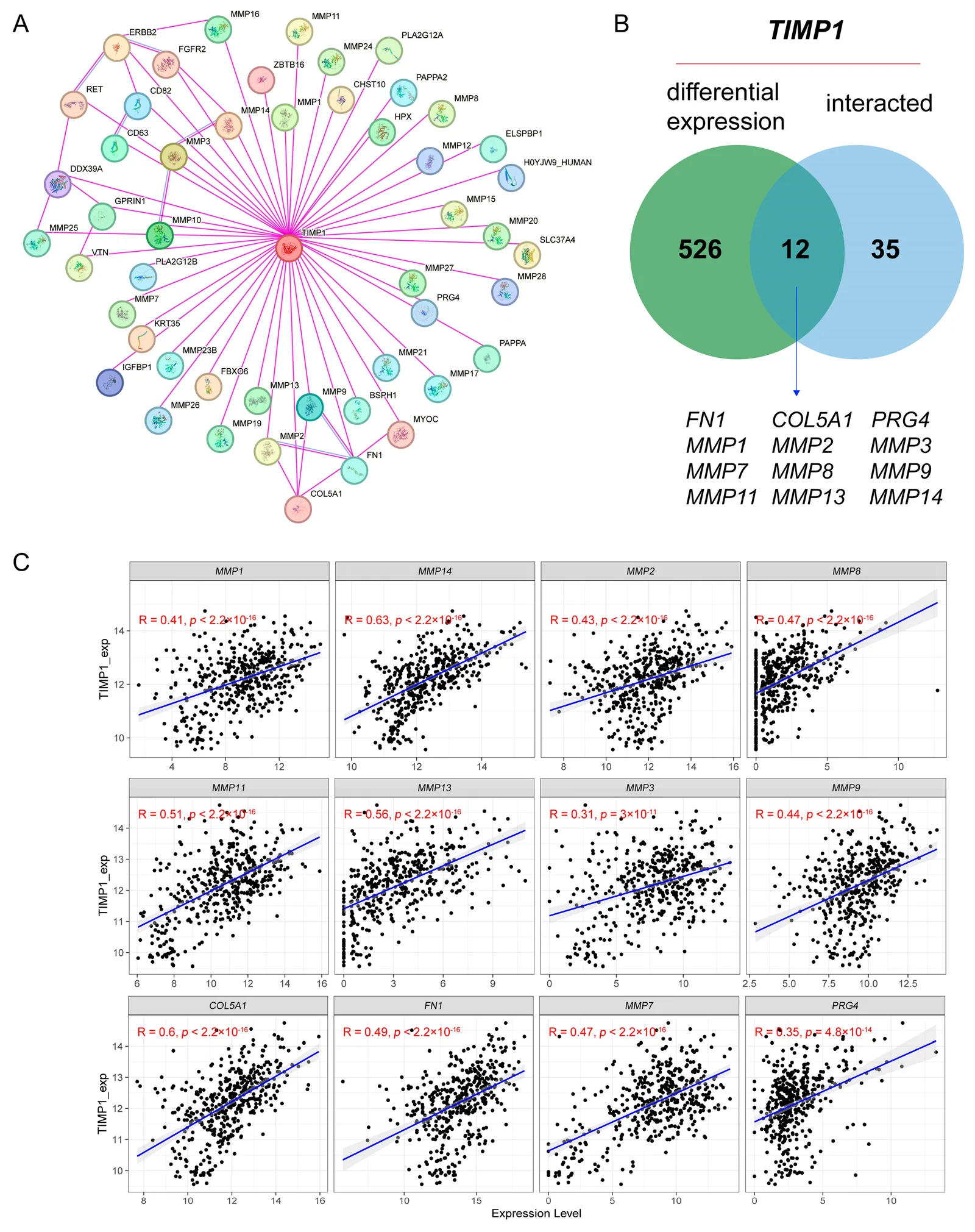
PPI network and core gene identification. (**A**) Network visualization of *TIMP1*-correlated genes. (**B**) A Venn-scheme illustration displaying shared candidates among *TIMP1*-binding protein partners and differentially expressed transcripts linked to this gene. (**C**) Expression correlations of *TIMP1* with the overlapping candidates.

The PPI network analysis identified 49 *TIMP1*-interacting proteins (Figure 8A). Overlap analysis identified 12 genes shared between *TIMP1*-interacting proteins and *TIMP1*-associated DEGs: *COL5A1*, *FN1*, *PRG4*, and a cluster of nine *MMPs* (*MMP1*/*2*/*3*/*7*/*8*/*9*/*11*/*13*/*14*) (Figure 8B). Spearman’s correlation analysis confirmed significant positive correlations between *TIMP1* and all 12 genes (r ranging from 0.31 to 0.63, all *p* < $2.2\times 10^{-16}$) (Figure 8C).

## 4. Discussion

### 4.1. Key Findings

Through an integrative strategy combining bioinformatic predictions with experimental expression validation, we systematically characterized the transcriptional landscape, clinical prognostic value, and immune-related associations of *TIMP1* in CRC. Notably, CRC tissues and cell lines exhibited pronounced *TIMP1* overexpression, and this elevated abundance was inversely linked to both overall and disease-specific survival. It should be noted that the AUC values for *TIMP1* (0.58–0.65) indicate only moderate predictive accuracy, which is generally insufficient for clinical decision-making when using a single biomarker. This modest performance is not unexpected, as CRC is a highly heterogeneous disease and a single gene product cannot fully capture its complexity. Moreover, the prognostic value of *TIMP1* may be influenced by tumor stage and molecular subtypes, and our bulk transcriptomic data may dilute cell-type-specific effects. Therefore, rather than advocating *TIMP1* as an independent clinical tool, we propose its potential utility within multi-gene signatures or composite biomarker panels. Future studies integrating *TIMP1* with other parameters are warranted to improve predictive performance.

Meanwhile, high *TIMP1* expression may promote tumor progression by inhibiting immune cell infiltration and forming an immunosuppressive microenvironment. These findings confirm that the expression level of *TIMP1* can be used as a biomarker for the immune infiltration status of CRC patients, assisting in determining whether patients are suitable for immunotherapy.

### 4.2. Strengths and Limitations

A key strength of this study is its integrative multi-omics approach, combining large-scale database analyses with experimental validation via RT-qPCR, which enhances the robustness and translational relevance of our findings. The multi-database strategy minimizes cohort-specific biases and improves the generalizability of the prognostic conclusions. Additionally, the comprehensive characterization of *TIMP1*’s impact on the TIME-spanning immune infiltration, immune checkpoint expression, chemokine profiling, and ECM-related pathways provides a holistic view of its pleiotropic roles in CRC progression, beyond the conventional focus on MMP inhibition alone.

Several limitations warrant consideration. First, although our RT-qPCR validation confirmed *TIMP1* overexpression in CRC cell lines, the functional experiments were limited to a single cell line (RKO) and did not include in vivo animal models or patient-derived samples to validate the observed immune-modulatory effects. Second, the correlative evidence linking *TIMP1* to immune checkpoints and chemokines, while compelling, does not establish causality; mechanistic validation will therefore demand deliberate genetic or pharmacological perturbations in future experimental settings. Finally, single-cell resolution studies would be valuable to delineate the cellular sources and recipients of *TIMP1*-mediated signals.

### 4.3. Comparison with Similar Research

Our findings identify *TIMP1* as a factor closely associated with the CRC TIME, which is essential for tumor progression and immunotherapy response [19]. *TIMP1* expression showed close correlations with immune cell infiltration, TILs, immune checkpoint genes, and chemokines [20]. Specifically, *TIMP1*-high CRC tissues exhibited increased neutrophil infiltration, and decreased monocyte, mast cell, and B cell infiltration. Neutrophils drive intestinal inflammation and malignant transformation via reactive oxygen species, serine proteases, MMPs, myeloperoxidases, and neutrophil extracellular trap formation [21,22,23,24], whereas monocytes exert context-dependent effects by differentiating into pro-tumor M2 macrophages (promoting growth, metastasis, and immune evasion [25,26,27]) or anti-tumor M1 macrophages/dendritic cells [28]. In contrast, mast and B cells play tumor-suppressive roles in CRC [29,30,31]. However, it should be noted that these infiltration patterns may be influenced by tumor purity and stromal content, as *TIMP1* expression positively correlates with stromal score and inversely with tumor purity. The apparent discrepancies between group-wise comparisons and correlation analyses likely reflect both methodological differences across algorithms and the confounding effects of stromal components. Therefore, our data point to a link between *TIMP1* abundance and immune infiltration profiles, though further studies with single-cell resolution or purified cell populations are needed to validate these associations. Collectively, these observations indicate that *TIMP1* shapes the pro-tumorigenic TIME by enhancing pro-tumor immune cell recruitment and attenuating anti-tumor immune cell infiltration.

Within the TIME, TILs play dual roles in tumor progression; they can either restrain or facilitate tumor growth alongside regulating immune surveillance. Our data demonstrated that *TIMP1* expression significantly correlated with diverse TILs and multiple immune checkpoint genes, supporting its potential as a central regulator of CRC immune responses that influence immune cell functionality via multi-faceted pathways. Notably, *TIMP1* correlates with key immune checkpoint molecules with well-established roles in CRC immunotherapy: *CD274* (PD-L1), for which up-regulation predicts anti-PD-1/PD-L1 response [19] and down-regulation enhances T cell activity in tumors [32]; CTLA-4, whose expression was markedly up-regulated following chemotherapy treatment [33]; TIGIT, an emerging target modulating checkpoint blockade responses [34]; and IDO1, a mediator of immune tolerance linked to CRC immune evasion, poor prognosis, and liver metastasis [35,36]. These associations, together with the correlation of *TIMP1* with other checkpoint genes implicated in CRC immunotherapy, collectively indicate that *TIMP1* participates in the remodeling of the TIME by regulating the activity of TILs, abundance, and functionality.

Additionally, *TIMP1* correlated with immunomodulatory chemokine genes (*CCL3, CCL5, CXCL8*, and *CXCL10*) that regulate immune cell trafficking and activation [37,38,39,40]. For example, via activation of the TRAF6/NF-κB pathway, *CCL3* accelerates proliferation and invasion in colorectal-cancer cells [37]; meanwhile, *CXCL10* augments CD8^+^ T cell infiltration and contributes to the normalization of tumor vasculature [40]. Therefore, *TIMP1* may modulate immune cell dynamics in the TIME by regulating chemokine expression.

Functional annotation of *TIMP1*-associated DEGs highlighted significant enrichment in ECM-receptor interactions, focal adhesion pathways, and PI3K-Akt signaling, all of which are critical for CRC progression and metastasis [41,42]. Twelve core interacting genes (*FN1*, *COL5A1*, *PRG4,* and nine MMPs) were involved in ECM composition, degradation, and remodeling. *FN1* and *COL5A1* are major ECM components that mediate cell adhesion and migration [43,44], whereas MMPs are key enzymes involved in ECM degradation and tumor invasion [5]. As a natural inhibitor of MMP, *TIMP1* maintains homeostasis of MMP activity [5], and our data showed positive correlations between *TIMP1* and multiple MMPs, suggesting a complex regulatory network in which *TIMP1* may fine-tune ECM remodeling to promote CRC progression. This is consistent with previous reports that *TIMP1* facilitates CRC metastasis via ECM remodeling [12].

### 4.4. Implications and Actions Needed

Collectively, our results may offer insights that are of potential relevance to both mechanistic exploration and clinical decision-making. The significant association between *TIMP1* expression and patient survival, together with its reported detectability in serum, positions *TIMP1* as a candidate non-invasive prognostic marker for CRC. Additionally, the observed correlations between *TIMP1* expression and immune checkpoint genes, along with its link to immune infiltration, suggest that *TIMP1* may warrant further investigation as a potential marker for immunotherapy-related studies.

However, the successful translation of biomarkers into clinical practice remains challenging. As reviewed by Malerba et al., artificial intelligence is emerging as a bridge connecting molecular discoveries with clinical decision-making in gastrointestinal cancers [45]. Concurrently, gut microbial metabolites such as sodium butyrate and nifedipine have attracted increasing attention for their protective effects against CRC, including the induction of cancer cell apoptosis, enhancement of therapy sensitivity, and protection of healthy cells [46,47]. These complementary perspectives, one technological and one biological, underscore the need for multi-dimensional approaches that integrate molecular biomarkers, AI-based predictive models, and microbiota-targeted interventions to advance precision medicine in CRC.

## 5. Conclusions

In summary, our data indicate that *TIMP1*, which is overexpressed in CRC and linked to potential poorer survival outcomes, may contribute to tumor progression through two convergent routes: modulation of immune infiltration (involving TILs, checkpoints, and chemokines) and interplay with ECM-related genes. These observations, while preliminary, imply that *TIMP1* warrants further evaluation as a potential prognostic indicator and as a candidate for guiding individualized treatment approaches.

## Data Availability

All data were derived from publicly available databases (TCGA and GEO).

## Supplementary Materials

The following supporting information can be downloaded at: https://www.mdpi.com/article/10.3390/genes17080977/s1, Figure S1. Differences in *TIMP1* expression among different T stages and pathological stages. (A) T1 + T2 vs. T3 + T4 stages. (B) Different pathological stages.

## Abbreviations

The abbreviations and their corresponding full terms are listed below:

CRC	Colorectal Cancer
NK	Natural Killer Cell
ECM	Extracellular Matrix
*TIMP1*	Tissue Inhibitor of Metalloproteinase 1
MMPs	Matrix Metalloproteinases
DEGs	Differentially Expressed Genes
ROC	Receiver Operating Characteristic
AUC	Area Under the Curve
KEGG	Kyoto Encyclopedia of Genes and Genomes
GO	Gene Ontology
HR	Hazard Ratio
TILs	Tumor-Infiltrating Lymphocytes
PPI	Protein–Protein Interaction Networks
OS	Overall Survival
DSS	Disease-Specific Survival
STRING	Search Tool for the Retrieval of Interacting Genes/Proteins
TISIDB	Tumor Immune System Interactions Database
