# Supplementary material for: Integrated Analysis of Multiple Databases Identifies Tissue Inhibitor of Metalloproteinase 1 Expression and Its Association with the Immune Microenvironment in Colorectal Cancer
Source: Genes (Basel). 2026 Aug 20;17(8):977. doi: 10.3390/genes17080977 (PMC13511886; doi:10.3390/genes17080977)
Supplement: Supplementary file 1 [file genes-17-00977-s001.zip › genes-4459803-supplementary.pdf]

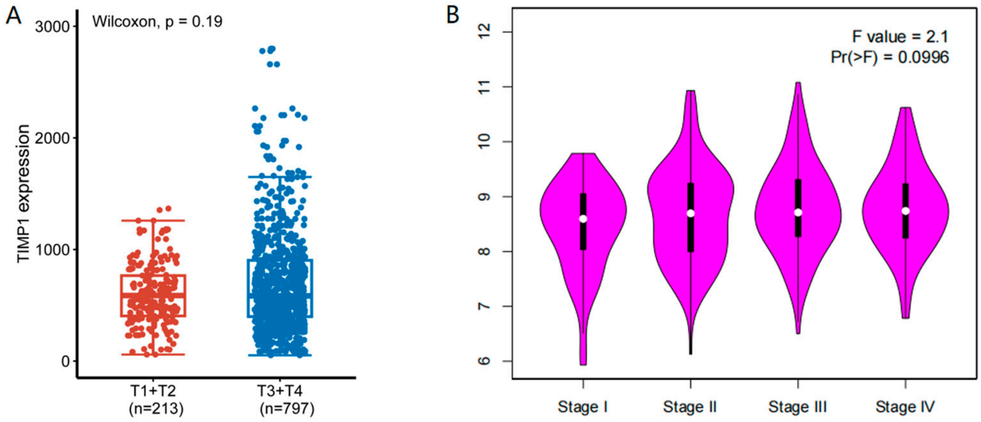

Figure S1. Differences in *TIMP1* expression among different T stages and pathological stages. (A) T1+T2 vs. T3+T4 stages. (B) Different pathological stages.
